# A COMPREHENSIVE DIGITAL SELF-CARE SUPPORT SYSTEM FOR OLDER ADULTS: A MULTIDISCIPLINARY FRAMEWORK

**DOI:** 10.1093/geroni/igz038.1190

**Published:** 2019-11-08

**Authors:** Priya Nambisan, Kalle Lyytinen, Kurt Stange, Eva Kahana, Gary L Kreps

**Affiliations:** 1 University of Wisconsin - Milwaukee, Milwaukee, Wisconsin, United States; 2 Case Western Reserve University, Cleveland, Ohio, United States; 3 George Mason University, Fairfax, Virginia, United States

## Abstract

This paper presents an innovative conceptual framework for designing a Comprehensive Digital Self-care Support System (CDSSS) to meet the health needs -physical, mental and social health needs of older adults and their caregivers. Older adults deal with multiple co-morbidities, medications and their side effects, fragmented care and often have poor understanding of their own health and treatments. These challenges call for solutions that lead to better empowerment and pro-active engagement and for support systems that focus on wellness and preventive care. The conceptual model we offer draws on diverse disciplines including health care management and medicine, information systems, communication, consumer behavior, and sociology to identify a set of key design principles for CDSSS. A review and analysis of the literature in the different fields led to the identification of 6 CDSSS design principles: (1) Systems approach; (2) User experience; (3) Ecosystem perspective for shared resources 4) Social and contextual learning; (5) Accessible design; (6) Designing for trust and empathy. The model clarifies how these design principles (or approaches) inform the development of the three main components of a CDSSS (data integration, communication, and resource integration) and enable the key CDSSS deliverables (learning, social & emotional support and care integration). The conceptual model also helps to lay out an agenda for future research on self-care support systems for older adults.

